# Type 1 diabetes mellitus: can coaching improve health outcomes?

**DOI:** 10.20945/2359-3997000000058

**Published:** 2018-08-01

**Authors:** Thais Pereira Costa Magalhães, Rodrigo Bastos Fóscolo, Aleida Nazareth Soares, Janice Sepúlveda Reis

**Affiliations:** 1 Instituto de Ensino e Pesquisa da Santa Casa de Belo Horizonte Instituto de Ensino e Pesquisa da Santa Casa de Belo Horizonte Belo Horizonte MG Brasil Instituto de Ensino e Pesquisa da Santa Casa de Belo Horizonte, Belo Horizonte, MG, Brasil; 2 Universidade Federal de Minas Gerais Universidade Federal de Minas Gerais Faculdade de Medicina Departamento de Clínica Médica Belo Horizonte MG Brasil Departamento de Clínica Médica, Faculdade de Medicina, Universidade Federal de Minas Gerais (UFMG), Belo Horizonte, MG, Brasil

**Keywords:** Type 1 diabetes mellitus, self-management, health coaching

## Abstract

**Objective:**

To evaluate the introduction of coaching in the interdisciplinary care of individuals with type 1 diabetes mellitus in the public health care system.

**Subjects and methods:**

Ten patients routinely attending a public health care service and with a glycated hemoglobin (HbA1c) level above 75% participated in eight coaching sessions. This study evaluated the patients' self-management of the disease and personal behavior. The participants were assessed at the beginning of the program and on two occasions after the intervention, with evaluation of biochemical and anthropometric data, and frequency of self-monitoring of blood glucose (SMBG). Questionnaires were applied during these evaluations to analyze emotional burden (B-PAID), medication adherence (Morisky Adherence Scale), and self-efficacy (IMDSES).

**Results:**

HbA1c had a median level of 8.0% (range 76-10.3%) at the beginning of the study and reduced significantly 3 months after initiation of the intervention (7.78% [6.5-10%], p = 0.028), with no significant increase at 6 months (8.3% [713-9.27%], p = 0.386). SMBG improved significantly from the beginning to the end of the study, with the median number of glucose tests per week varying from 16.5 (range 0-42) at baseline to 29.0 (7-42) at 3 months and 27.5 (10-48) at 6 months (p = 0.047). No significant differences were observed in anthropometric parameters or in the scores of the instruments between the three measurements.

**Conclusion:**

A coaching intervention focused on patients' values and sense of purpose may provide added benefit to traditional diabetes education programs and could be an auxiliary method to help individuals with type 1 diabetes achieve their treatment goals.

## INTRODUCTION

Treatment of type 1 diabetes (T1DM) requires several daily actions in pursuit of goals like the application of multiple daily doses of insulin, blood glucose monitoring, and regular physical activity. Despite many treatment advances, more than 70% of the individuals with T1DM maintain glycated hemoglobin (HbA1c) levels above 7% (1). Rates of treatment nonadherence for patients with diabetes often exceed 50%, emphasizing a need for interventions focused on behavioral change (2).

Coaching is a method that has proven useful in enhancing personal insight, and has received special attention as a method to improve healthy lifestyle behaviors (3). Health coaching is “a practice of health education and health promotion within a coaching context to enhance the well-being of individuals, and facilitate the achievement of their health-related goals” (4). It is distinct from other diabetes education strategies in that the patient is encouraged to choose goals that are aligned with his or her values.

To the best of our knowledge, no information is available about coaching as a strategy for diabetes education in Brazil, or about the most appropriate tools of the coaching process in clinical practice. Based on that, the aim of this study was to evaluate the introduction of coaching to the interdisciplinary care of individuals with T1DM in the public health care system.

## SUBJECTS AND METHODS

This was a pilot, longitudinal study including 10 patients with T1DM on a basal-bolus (NPH or glargine and lispro) insulin regimen, with a minimum of 50% of the total bolus dose, and with inadequate glycemic control (HbA1c levels ≥ 7.5%). The patients received care from an interdisciplinary team comprising endocrinologists, nutritionists, nurses, physical educators, and psychologists for at least 1 year at the Santa Casa Hospital, a public health care center located in Belo Horizonte (Minas Gerais, Brazil). The study protocol was approved by the institution's Ethics Committee and written informed consent for participation in the study was obtained from all volunteers. Exclusion criteria included cognitive impairment, pregnancy, and visual deficit.

### Intervention

The intervention was delivered by a single coach, an endocrinologist with substantial training in coaching methods. The participants were offered weekly 60-minute individual coaching sessions for a total of eight sessions, established according to standardized method interventions that varied from 5 to 14 (5,6). During this period, no additional intervention was performed by the interdisciplinary team. The patients were evaluated before the intervention and at 3 and 6 months thereafter.

**Figure 1:**
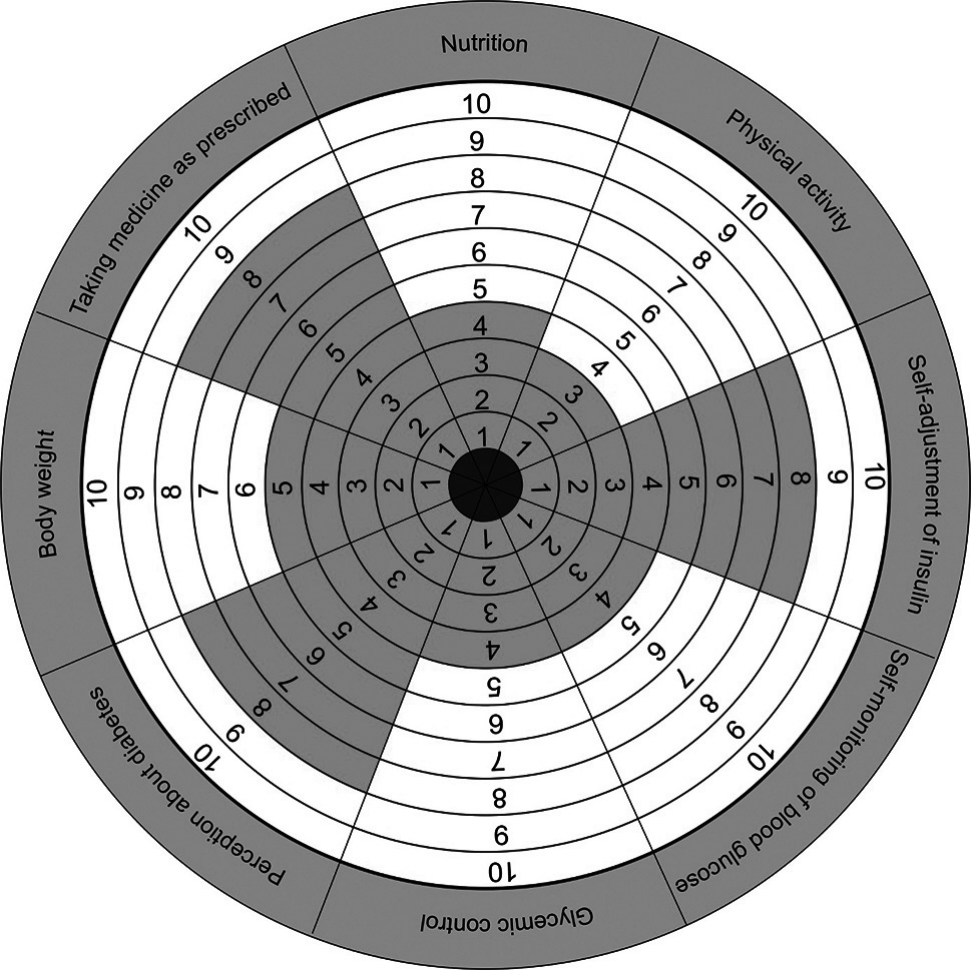
Wheel of self-care in diabetes filled out by one of the participants. Note that physical activity was the area that received the lowest score, followed by self-monitoring of blood glucose, glycemic control, and nutrition. The areas received different scores, resulting in a rather irregular wheel due to treatment imbalance.

The main components of a method that has been previously published in studies about coaching and health were applied in this study (5,7) (Table 1). In an analogy to the Wheel of Health (8), a new tool was developed, named the Wheel of Self-Care in Diabetes (Figure 1), which is divided into eight dimensions of diabetes treatment. The participant scored each area on a scale of 0-10 points, with 0 meaning “no attention given to the dimension” and 10 meaning “total attention given to the dimension”.

**Table 1:** Principles and tools of coaching

Principles of coaching
***Focus on the future***
It focuses on the solution to problems rather than their source. A short, medium or long-term goal is defined.
***Action***
It is performed by weekly tasks, which are defined at the end of each session and must have deadlines to start and finish.
***Autonomy***
The goals to be achieved at the end of the process, as well as the weekly tasks and deadlines, are defined by the individuals in coaching, who have full autonomy in their choices.
***Active listening without judgment***
Building a relationship of trust in which the client can express himself freely.
***Effective questions***
These are open-ended questions that stimulate reflection and the elaboration of responses directed to new possibilities in the face of obstacles and difficulties. In addition, it generates the person's commitment to his own speech and decisions.
***Focus on the positive***
It seeks the optimistic look on adversity, seeking to resignify bad events and bringing to light more positive alternatives for confronting problems.
**Coaching tools**
***S.M.A.R.T.***
Once the goal is set, this tool is applied to format it, making the goal more realistic and achievable: S - specific; M - mensurable; A - attainable; R - relevant; T - time.
***Behavioral profile***
Questionnaire whose result indicates to the clients which behavior prevails in their day-to-day attitudes (analyst, communicative, executive, idealizer) and discusses how the predominant behavior helps or hinders the achievement of their goals.
***Wheel of life***
This tool was administered during the initial assessment to help guide the conversation, with participants reporting how successful or satisfied they were (0-10) in each life domain (career, family, financial, spiritual, health, intellectual, among others). This is a clinical tool to explore values, establish priorities, and set goals. Identifying areas in which the participants felt less successful or satisfied, they then chose areas on which to focus for coaching.
***Action plan***
It involves defining the first steps towards the goal, the goals to be achieved and contributing to the ultimate goal, predicting obstacles and how to circumvent them, and recruiting the skills and competencies needed to reach the desired state.

The eight sessions delivered followed the format: Session 1 - understanding the behavioral profile and defining the participant's current state using the Wheel of Life and Wheel of Self-Care in Diabetes; Sessions 2 and 3 - definition of the desired status, choice and detail of the objectives, and desired results with the intervention. In order to reach the goal, small steps were defined towards the desired result at the end of all sessions, and participants demonstrated their accomplishment through photos, text messages, e-mails, and letters. The tasks were chosen by the participants themselves, who determined how and when to execute them. For example, if the goal was to achieve better glycemic control, the participant could define as a task an adjustment in diet or accomplishment of an increased number of capillary glucose testing per day; Sessions 4 to 8 - planning and execution of these actions, and choosing new goals for the future.

### Outcome variables

Values of HbA1c, body mass index (BMI), total daily insulin dose (TDD), lipid profile, blood pressure, and frequency of self-monitoring of blood glucose (SMBG) were evaluated during the three measurement sessions. The following validated surveys, which have demonstrated adequate psychometric properties, were applied to the participants: Problem Areas in Diabetes, Brazilian version (B-PAID) (9), which assesses the emotional overload related to diabetes (the results range from 0-100 points, with scores equal to or above 40 points indicating a high level of emotional distress); Insulin Management Diabetes Self-Efficacy Scale (IMDSES) (10), which evaluates self-efficacy (the results range from 28-112 points, with a lower score reflecting increased self-effectiveness); and the Morisky Adherence Scale (11), which evaluates the patient's adherence to medication use (based on the results, the adhesion is considered to be high [8 points], medium [6 to 8 points], or low [< 6 points]).

### Data analysis

Statistical analyses were performed using SPSS, v.20.0 (Chicago, Illinois, USA). Continuous variables are described with measures of central tendency (mean and median), standard deviation, and range (minimum and maximum values). Wilcoxon signed-rank tests were used for nonnormally distributed data. Statistical significance was set at 0.05 for each test.

## RESULTS

The subjects comprised mostly women (60%), had a mean age of 30 ± 8 years, diabetes duration of 13.0 ± 6.4 years, education level of high school or more (90%), and a family income of 3-4 monthly minimum wages (60%). The participants presented a mean BMI of 25.3 ± 5.5 kg/m^2^, mean systolic (SBP) and diastolic (DBP) blood pressure levels of 122.0 ± 10.3 and 81.0 ± 9.9 mmHg, respectively, and serum levels of total cholesterol of 168.3 ± 44.5 mg/dL, LDL-cholesterol 91.1 ± 41.2, HDL-cholesterol 58.7 ± 18.6 mg/dL, triglycerides 71.3 ± 21.0 mg/dL, and TDD 0.92 ± 0.53 UI/kg/day. No significant differences were observed between the values of BMI, SBP, DBP, lipids, and TDD across the three measurement sessions (0, 3, and 6 months).

HbA1c had a median level of 8.0% (7.6 – 10.3%) at the beginning of the study, which reduced significantly 3 months after the beginning of the intervention (7.78% [6.5-10%], p = 0.028), with no significant increase at 6 months (8.3% [7.13-9.27%], p = 0.386). There was a significant improvement in SMBG from the beginning to the end of the study, with the number of glucose measurements per week ranging from a median of 16.5 (0-42) at baseline, to 29.0 (7-42) at 3 months and 27.5 (10-48) at 6 months (p = 0.047).

Regarding the instruments, the median scores of the B-PAID showed a low emotional overload at the beginning of the study (21 [6-54]) and no significant difference at 3 months (17 [10-56]) and 6 months (13 [4-49]), p > 0.05). The IMDSES, which evaluates self-efficacy, demonstrated increased self-efficacy at the beginning of the study (median scores 41 [26-52]) and no significant difference at 3 months (35 [25-51]) and 6 months (36 [22-52], p > 0.05). The Morisky Scale scores showed an average adherence rate at the beginning of the study (median score 6 [5-7]), without significant changes at 3 (7 [4-7]) and 6 months (7 [37], p > 0.05).

## DISCUSSION

To the best of our knowledge, this is the first study conducted in the public health care system in Brazil to analyze the effectiveness of an individualized diabetes coaching intervention in addition to providing education to T1DM patients. The results of the study demonstrated that coaching is a method that can contribute to the achievement of glycemic control in these patients.

The participants in this study had already received guidance on aspects related to the disease and its treatment. It is in this scenario that coaching favors the patients, who by becoming informed, are able to define their goals, encouraged to choose the deadlines to initiate the changes, and become more participative and autonomous in their decisions by putting their knowledge into practice (12,13).

Patients are generally used to following prescriptions and recommendations by health care professionals. In coaching, patients are provided with new insights into how they can approach treatment, with independence and self-responsibility, establishing how and when changes are made (14), with greater dedication to performing daily activities related to diabetes care.

The ten participants appreciated the method, were encouraged by the proposal of autonomy in relation to treatment decisions, and concluded the tasks they set out to carry out, as demonstrated in the statements: “Coaching brought several changes to my treatment, such as dose adjustment and time of insulin application. Looking at the Wheel of Self-Care in Diabetes, I realized the attention that I was giving to each aspect of my treatment and it was a surprise to me” (ECGP, 29); “Choosing the time, date, and place to accomplish the tasks (goals) and still have to prove that I performed them helped me a lot. Before, everything was only planned. I wanted to do it, but I could not” (FAVS, 43).

This study demonstrated a decrease in HbA1c levels and an increase in the frequency of SMBG, with no changes in TDD, which can be explained by greater commitment and motivation to perform the various actions necessary to improve metabolic control, such as corrections of hyperglycemia and appropriate treatment of hypoglycemia. Studies in the literature have also found favorable results for coaching in the approach of individuals with diabetes, such as the improvement of HbA1c levels and quality of life (6-8). Other studies have shown that the results are time-dependent, or, the longer the coaching process, the better the responses to quality of life, drug compliance, and self-efficacy (8,12,15). In this study, we observed no significant improvement in these three aspects. The fact that the participants had already a good score on the instruments at baseline can be justified by interdisciplinary care and long-term diabetes education programs, and explains little changes during the study.

Although coaching is a heterogeneous intervention, it may be applied to T1DM patients in the context of the public health care service, as long as its fundamental characteristics are present: establishing goals and objectives, and ensuring patient autonomy in the process and action through tasks. Thus, in the day-to-day care of individuals with T1DM, coaching can be inserted formally by a qualified professional using the method integrally with its tools and techniques, or informally by other team members during routine appointments or as part of education programs. The various tools of coaching, especially the Wheel of Self-Care in Diabetes, developed for the purpose of this study, can be inserted routinely in education programs, serving as a starting point for reflection and setting goals/objectives. The formal approach should consist of programmed sessions, and the informal approach can occur whenever necessary or ongoing throughout the patient follow-up.

In conclusion, a coaching intervention focused on the patient's values and sense of purpose may provide added benefit to traditional diabetes education programs. Fundamentals of coaching may be applied by diabetes educators to improve patient self-efficacy, accountability, and clinical outcomes. New studies are needed, with a larger number of participants, in order to expand the use of the coaching methodology within the interdisciplinary treatment of diabetes.

## References

[1] Gomes MB, Coral M, Cobas RA, Dib SA, Canani LH, Nery M, et al Prevalence of adults with type 1 diabetes who meet the goals of care in daily clinical practice: a nationwide multicenter study in Brazil. Diabetes Res Clin Pract. 2012;97(1):63-70. 10.1016/j.diabres.2012.02.008 22397904

[2] Dean AJ, Walters J, Hall A. A systematic review of interventions to enhance medication adherence in children and adolescents with chronic illness. Arch Dis Child. 2010;95(9):717-23. 10.1136/adc.2009.175125 20522463

[3] Olsen JM, Nesbitt BJ. Health coaching to improve healthy lifestyle behaviors: an integrative review. Am J Health Promot. 2010;25(1):e1-e12. 10.4278/ajhp.090313-LIT-101 20809820

[4] Palmer S, Tubbs I, Whybrow A. Health coaching to facilitate the promotion of healthy behaviour and achievement of health-related goals. Int J Heal Promot Educ. 2003;41(3):91-3.

[5] Wong-Rieger D, Rieger FP. Health coaching in diabetes: empowering patients to self-manage. Can J diabetes. 2013;37(1):41-4. 10.1016/j.jcjd.2013.01.001 24070747

[6] Ammentorp J, Uhrenfeldt L, Angel F, Ehrensvärd M, Carlsen EB, Kofoed P-E. Can life coaching improve health outcomes? - A systematic review of intervention studies. BMC Health Serv Res. 2013;13(1):428. 10.1186/1472-6963-13-428 PMC4015179 24148189

[7] Ammentorp J, Thomsen J, Kofoed P-E. Adolescents with Poorly Controlled Type 1 Diabetes can Benefit from Coaching: A Case Report and Discussion. J Clin Psychol Med Settings. 2013;20(3):343-50. 10.1007/s10880-013-9374-z 23900748

[8] Wolever RQ, Dreusicke M, Fikkan J, Hawkins TV, Yeung S, Wakefield J, et al. Integrative health coaching for patients with type 2 diabetes: a randomized clinical trial. Diabetes Educ. 2010;36(4):629-39. 10.1177/0145721710371523 20534872

[9] Gross CC, Scain SF, Scheffel R, Gross JL, Hutz CS. Brazilian version of the Problem Areas in Diabetes Scale (B-PAID): Validation and identification of individuals at high risk for emotional distress. Diabetes Res Clin Pract. 2007;76(3):455-59. 10.1016/j.diabres.2006.09.022 17081645

[10] Gastal DA, Pinheiro RT, Vazquez DP. Self-efficacy scale for Brazilians with type 1 diabetes. Sao Paulo Med J. 2007;125(2):96-101. 10.1590/S1516-31802007000200006 PMC11014699 17625707

[11] Ben AJ, Neumann CR, Mengue SS. The Brief Medication Questionnaire and Morisky-Green test to evaluate medication adherence. Rev Saude Publica. 2012;46(2):279-89. 10.1590/s0034-89102012005000013 22331180

[12] Nishita C, Cardazone G, Uehara DL, Tom T. Empowered diabetes management: life coaching and pharmacist counseling for employed adults with diabetes. Health Educ Behav. 2013;40(5):581-91. 10.1177/1090198112465088 23174629

[13] Raddock M, Martukovich R, Berko E, Reyes CD, Werner JJ. 7 tools to help patients adopt healthier behaviors. J Fam Pract. 2015;64(2):97-103. 25671537

[14] Koenigsberg MR, Bartlett D, Cramer JS. Facilitating treatment adherence with lifestyle changes in diabetes. Am Fam Physician. 2004;69(2):309-16. 14765768

[15] Glasgow RE, La Chance PA, Toobert DJ, Brown J, Hampson SE, Riddle MC. Long-term effects and costs of brief behavioural dietary intervention for patients with diabetes delivered from the medical office. Patient Educ Couns. 1997;32(3):175-84. 10.1016/s0738-3991(97)00039-6 9423499

